# Sodium/glucose cotransporter 2 is expressed in choroid plexus epithelial cells and ependymal cells in human and mouse brains

**DOI:** 10.1111/neup.12665

**Published:** 2020-06-02

**Authors:** Yoichi Chiba, Yasunori Sugiyama, Nozomu Nishi, Wakako Nonaka, Ryuta Murakami, Masaki Ueno

**Affiliations:** ^1^ Department of Pathology and Host Defense Kagawa University Kagawa Japan; ^2^ Department of Life Sciences Kagawa University Kagawa Japan; ^3^ Life Science Research Center Kagawa University Kagawa Japan; ^4^ Department of Supportive and Promotive Medicine of the Municipal Hospital Kagawa University Kagawa Japan; ^5^ Department of Gastroenterology and Neurology Kagawa University Kagawa Japan

**Keywords:** central nervous system, diabetes mellitus, immunolabeling, mRNA, SLC5A2

## Abstract

Diabetes mellitus (DM) is now recognized as one of the risk factors for Alzheimer's disease (AD), and the disease‐modifying effects of anti‐diabetic drugs on AD have recently been attracting great attention. Sodium/glucose cotransporter 2 (SGLT2) inhibitors are a new class of anti‐diabetic drugs targeting the SGLT2/solute carrier family 5 member 2 (SLC5A2) protein, which is known to localize exclusively in the brush border membrane of early proximal tubules in the kidney. However, recent data suggest that it is also expressed in other tissues. In the present study, we investigated the expression of SGLT2/SLC5A2 in human and mouse brains. Immunohistochemical staining of paraffin sections from autopsied human brains and C3H/He mouse brains revealed granular cytoplasmic immunoreactivity in choroid plexus epithelial cells and ependymal cells. Immunoblot analysis of the membrane fraction of mouse choroid plexus showed distinct immunoreactive bands at 70 and 26 kDa. Band patterns around 70 kDa in the membrane fraction of the choroid plexus were different from those in the kidney. Reverse transcription‐polymerase chain reaction analysis confirmed the expression of *Slc5a2* mRNA in the mouse choroid plexus. Our results provide *in vivo* evidence that SGLT2/SLC5A2 is expressed in cells facing the cerebrospinal fluid, in addition to early proximal tubular epithelial cells. These findings suggest that SGLT2 inhibitors may have another site of action in the brain. The effects of SGLT2 inhibitors on brain function and AD progression merit further investigation to develop better treatment options for DM patients.

## INTRODUCTION

Accumulating epidemiological evidence supports a link between diabetes mellitus (DM) and Alzheimer's disease (AD). Systematic reviews and meta‐analyses have presented the relative risk for AD in diabetic patients compared to nondiabetic patients as 1.53 (95% CI 1.42–1.63).^1^ Although the majority of the autopsy studies report no association between type 2 DM (T2DM) and the burden of amyloid plaque or neurofibrillary tangles, which are the neuropathological hallmarks of AD,^2^ the biochemical mechanisms upstream of early AD‐specific pathology may overlap with those of T2DM.^2^, ^3^ Shared upstream pathways between AD and T2DM include cerebral and systemic insulin resistance, vascular endothelial dysfunction, mitochondrial dysregulation, oxidative stress, accumulation of advanced glycation end products, neuroinflammation, and blood–brain barrier dysfunction.^2^, ^3^, ^4^, ^5^ Given the multiple links between AD and T2DM, it is plausible that anti‐diabetic drugs could be beneficial to modify the progression of AD. The effects of several anti‐diabetic drugs on the central nervous system (CNS) have recently been reviewed.^4^, ^5^ A recent meta‐analysis revealed that the use of pioglitazone, a potent and selective agonist of peroxisome proliferator‐activated receptor‐γ, reduced the risk of dementia by 47%.^6^

Sodium‐glucose cotransporter (SGLT) 2 inhibitors (SGLT2‐Is) are a new class of anti‐diabetic drugs and are recommended as a second antihyperglycemic agent for patients with T2DM who have complications like atherosclerotic cardiovascular disease, heart failure, or chronic kidney disease.^7^, ^8^ Besides their antihyperglycemic action, SGLT2‐Is also have a range of *in vivo* effects involving hemodynamic and metabolic pathways.^7^ The effects of SGLT2‐Is on AD progression have not yet been studied,^5^ although certain drugs (i.e., dapagliflozin and canagliflozin) might act as potent dual inhibitors of SGLT2 and acetylcholinesterase (AChE), implying that SGLT2‐Is may have some therapeutic potential for DM‐associated AD.^9^, ^10^

SGLT2/SLC5A2 protein is reported to be exclusively expressed on the brush border membrane (BBM) of proximal tubular epithelial (PTE) cells in the kidney.^11^, ^12^ Notably, Bonner *et al*. (2005) reported SGLT2/SLC5A2 expression in the α cells of the human pancreatic islets.^13^ In the CNS, elevated expression of SGLT2/SLC5A2 protein has been reported in diseased tissues, such as in high‐grade astrocytoma^14^ and traumatic brain injury.^15^ Furthermore, the expression of SGLT2/SLC5A2 mRNA, encoding SGLT2/SLC5A2 protein, has been reported in the cerebellum, and at low levels in the heart, salivary gland, liver, and thyroid gland.^16^ These data suggest that SGLT2/SLC5A2 may be expressed in tissues other than the kidney, and that SGLT2‐Is may have unexpected effects other than antihyperglycemic action.

Ependymal cells are epithelium‐like glial cells lining the ventricular system of the CNS, and those covering the choroid plexus specialize as cerebrospinal fluid (CSF)‐producing choroid plexus epithelial (CPE) cells. CPE cells express many solute carrier (SLC) transporters that facilitate the transport of organic molecules as well as inorganic ions. Of interest, several SLC transporters for monosaccharides and uric acid, including glucose transporter (GLUT) 1 (GLUT1), GLUT5, GLUT8, GLUT9, and urate transporter 1 (URAT1), are commonly expressed in CPE and PTE cells.^17^, ^18^, ^19^, ^20^, ^21^

Given the expression of SGLT2/SLC5A2 mRNA in tissues other than the kidney and given the similarity in the expression profile of SLCs between CPE and PTE cells, we sought to determine whether SGLT2/SLC5A2 mRNA was also expressed in CPE cells, using reverse transcription (RT)‐poymerase chain reaction (PCR) analysis. In this study, we report the expression of SGLT2/SLC5A2 in CPE and ependymal cells in human and mouse brains. Our results suggest additional physiological roles of SGLT2/SLC5A2 in cells exposed to the CSF and the presence of a new potential target of SGLT2‐Is in the brain.

## MATERIALS AND METHODS

### Human tissues

Human tissue samples were obtained at autopsy in Kagawa University Hospital, as described previously.^19^, ^22^ Table 1 summarizes the clinical profiles of all subjects examined. This study was approved by the institutional Ethics Committee of the Faculty of Medicine, Kagawa University (#H24‐48) in accordance with the Declaration of Helsinki, and was performed with the written informed consent of all relevant persons received prior to the subject inclusion. After fixation in 10% formalin, paraffin‐embedded tissue blocks were prepared and cut into 4‐μm‐thick sections.

**Table 1:** Clinical profiles of the subjects

Case No.	Age/sex	Main diagnosis	PMD (h)	DM	HbA1c (%)	Duration of DM (y)
1	30–39/F	Brainstem hemorrhage	2.5	–	N/A	N/A
2	70–79/M	Cerebellar tuberculosis	1	Type 2	9.6	33
3	60–69/M	Dissecting aneurysm	5	+ (Type unknown)	12.3	N/A
4	60–69/F	Thalamic hemorrhage	5	–	N/A	N/A
5	70–79/F	Pneumonia	4	–	N/A	N/A
6	70–79/M	Unstable angina	3	Type 2	6.7	N/A

DM, diabetes mellitus; F, female: h, hour(s); HbA1c, last hemoglobin A1c value (NGSP) before death; M, male; N/A, not available/not applicable; PMD, postmortem delay; y, years.

### Animals

All animal studies were approved by the Kagawa University Animal Care and Use Committee (#18624), and all efforts were made to minimize the number of and the extent of suffering of animals used. Under deep anesthesia with intraperitoneal injection of pentobarbital, 9‐week‐old male C3H/HeSlc mice (Japan SLC, Hamamatsu, Japan) were transcardially perfused with phosphate‐buffered saline (PBS), followed by the procedures described below.

### Immunohistochemistry

Paraffin‐embedded sections from the human kidney and medial temporal lobe of the cerebrum were deparaffinized, rehydrated, and pretreated with 0.3% hydrogen peroxide in PBS for 30 min to quench endogenous peroxidase activity. After blocking of nonspecific antibody binding with 2% bovine serum albumin (BSA) in PBS for 30 min, the sections were incubated with a rabbit polyclonal anti‐SGLT2/SLC5A2 antibody (1:400, NBP1‐92384, Novus Biologicals, Centennial, CO, USA), which specifically recognized amino acid residues 220–266 of human SGLT2/SLC5A2, at 4°C overnight. No antigen‐retrieval treatment was required. Sections were washed with PBS, and immunoreaction product deposits were visualized by the polymer‐immunocomplex method using a polymer solution conjugated with anti‐rabbit IgG and horseradish peroxidase (HRP) (Histofine Simple Stain MAX PO [R], Nichirei Biosciences, Tokyo, Japan) and developed with 3,3′‐diaminobenzidine. The sections were counterstained with hematoxylin. Control experiments were performed using the anti‐SGLT2/SLC5A2 antibody preincubated with 100‐fold molar excess of SGLT2/SLC5A2 recombinant protein antigen (NBP1‐92384PEP, Novus Biologicals) at 4°C overnight.

C3H/HeSlc mice (*n* = 3) were perfused with 4% paraformaldehyde in 0.1 M phosphate buffer (pH 7.4) following perfusion with PBS. Dissected tissues were postfixed with the same fixative at 4°C overnight, embedded in paraffin, and cut into 4‐μm‐thick sections. Immunohistochemical staining was performed as above, except that the antibody was diluted 1:200 for mouse tissues.

Paraffin‐embedded sections from human and mouse tissues were also immunostained a rabbit polyclonal with anti‐SGLT1/SLC5A1 antibody (2 μg/mL, ab14685, Abcam, Cambridge, UK), which specifically recognized amino acid residues 603–623 of human SGLT1/SLC5A1. After deparaffinization and endogenous peroxidase blocking, antigen retrieval was performed by heating sections in trishydroxy methyl amino methane (Tris)‐ethylenediaminetetraacetic acid (EDTA) buffer (pH 9.0) for 20 min, followed by blocking with 2% BSA. Immunostaining experiments were performed as above, except that the sections were incubated with the anti‐SGLT1/SLC5A1 antibody at room temperature for 1 h.

### Immunoblotting and densitometry

Membrane fractions of mouse tissues were prepared as described previously^19^ with some modifications. After perfusion with PBS, isolated mouse tissues were placed in cold PBS, and small pieces of tissues were dissected out and weighed. The tissues were homogenized in 14 volumes (v/w) of PBS containing protease inhibitor cocktail (PIC: Halt Protease Inhibitor Cocktail, Thermo Fisher Scientific, Waltham, MA, USA). Choroid plexus tissues were isolated from the lateral and fourth ventricles under a stereo microscope, placed in 10 μL of PBS containing PIC, and dissociated by triturating through a 200‐μL micropipette tip several times followed by vigorously mixing with a vortex mixer. After centrifuging at 21 500 *g* at 4°C for 30 min, the resulting pellets were resuspended in 14 volumes (v/w) of PBS containing PIC and used as the membrane fraction. The protein concentration was determined using a Pierce BCA Protein Assay Kit (Thermo Fisher Scientific) following the addition of nine volumes of 50 mM sodium hydroxide (NaOH) to each sample.

The membrane fraction was solubilized in sodium dodecyl sulfate (SDS) sample buffer by heating at 95°C for 3 min, and 10 μg of protein as each aliquot was subjected to SDS‐polyacrylamide gel electrophoresis (PAGE) on a 10% polyacrylamide gel along with a molecular mass marker solution (VisiMax Dual Marker Low, Cosmo, Tokyo, Japan). The separated proteins were transferred to polyvinylidene difluoride membranes (Wako Pure Chemical, Osaka, Japan) using the semi‐dry technique. The membranes were stained with Coomassie Brilliant Blue (CBB) (Rapid Stain CBB Kit, Nacalai Tesque, Kyoto, Japan) and scanned to quantify the amount of protein on blots. After destaining with a Rapid CBB Destain Kit (Nacalai Tesque), membranes were treated with 5% skim milk in Tris‐buffered saline containing 0.1% Tween 20 (TBST) to block nonspecific antibody binding, and then probed with the anti‐SGLT2/SLC5A2 antibody (1:500) at 4°C overnight. After washing with TBST, blots were incubated with HRP‐conjugated anti‐rabbit IgG (1:20 000, GE Healthcare, Buckinghamshire, UK). The primary and secondary antibodies were diluted in Can Get Signal Immunoreaction Enhancer Solution (Toyobo, Osaka, Japan). The immunoreactive bands were visualized using an ECL Western Blotting Analysis System (GE Healthcare) and a chemiluminescence imager (ImageQuant LAS4010: GE Healthcare). The density of immunoreactive signal bands was quantified using ImageJ (version 1.52a) and normalized with the amount of total blotted protein per lane assessed by CBB staining of the membranes. To demonstrate the specificity of the antibody, the diluted anti‐SGLT2/SLC5A2 antibody was preincubated with 24‐fold molar excess of SGLT2/SLC5A2 recombinant protein antigen at 4°C overnight.

### RT‐PCR

After perfusion with PBS, choroid plexus and kidney tissues were isolated from mice (n = 3), and total RNA was extracted using a ReliaPrep RNA Tissue Miniprep System (Promega, Fitchburg, WI, USA). The cDNA was synthesized with reverse transcriptase using a ReverTra Ace qPCR RT Master Mix (Toyobo). Ten nanograms of cDNA were used as a template, and the specific portions of the two transcripts were amplified with GoTaq G2 Hot Start Polymerase (Promega) and primer sets specific for the SGLT2/SLC5A2 gene (*Slc5a2*) and the glyceroaldehyde‐3‐phosphate dehydrogenase (GAPDH) gene (*Gapdh)* (Table 2). PCR amplification cycle conditions were as follows: (i) 2 min at 95C; (ii) 25, 30, or 35 cycles of 30 s at 95C, 15 s at 55C, and 30 s at 72C; and (iii) 5 min at 72°C. The amplicons were electrophoresed on a 2% agarose gel, stained with Midori Green Advance (Nippon Genetics, Tokyo, Japan), and visualized with a blue LED (470 nm) transilluminator (AMZ System Science, Osaka, Japan). Amplified fragments were subjected to direct sequencing (Eurofins Genomics, Tokyo, Japan).

**Table 2:** Primer sequences used for RT‐PCR

Gene	Primers (5′‐3′)	Location	Amplicon size (bp)
*Slc5a2*	F: CATTGGTGTTGGCTTGTGGT	102–121	111
	R: GCGAACAGAGAGGCTCCAAC	212–193	
*Gapdh*	F: CAAGGTCATCCATGACAACTTTG	527–549	496
	R: GTCCACCACCCTGTTGCTGTAG	1022–1001	

### Data presentation and statistical analysis

The densities of SGLT2/SLC5A2‐immunoreactive signal bands around 70 kDa were expressed as the ratio to the average value in the kidney samples from four mice and compared between kidney and choroid plexus using a paired *t*‐test. The data were analyzed with a GraphPad Prism (version 6.07, GraphPad Software, San Diego, CA, USA).

## RESULTS

### Validation of anti‐SGLT2/SLC5A2 antibody

**Figure 1:**
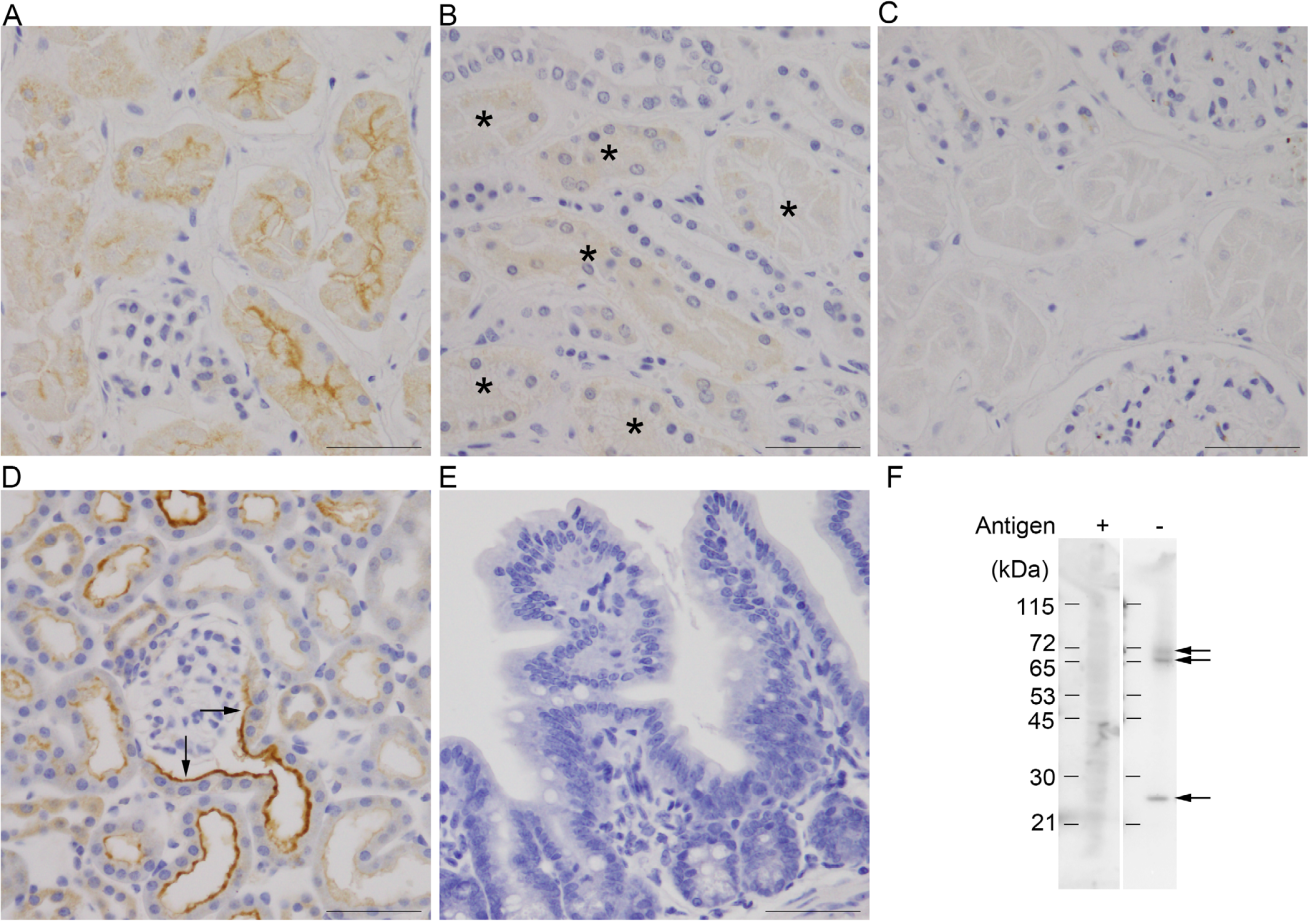
Validation of the specificity of anti‐sodium/glucose cotransporter 2 (SGLT2)/solute carrier family 5 member 2 antibody (SLC5A2) antibody used in the present study. (A, B) Immunohistochemical validation of the specificity of an anti‐SGLT2/SLC5A2 antibody by immunohistochemical (A‐E) and immunoblot (F) analyses using human (A‐C) and mouse (D‐F) tissues. (A) The human renal cortex containing glomerulus and early proximal convoluted tubules with the BBM is immunoreactive for SGLT2/SLC5A2. (B) The outer stripe containing the straight portion of proximal tubules (asterisks) exhibits no significant immunoreactivity on the BBM. (C) Preabsorption of the antibody with recombinant SGLT2/SLC5A2 protein antigen completely abolishes the immunoreactivity on the BBM of the early proximal tubules in human kidney. (D) In the mouse kidney tissue, in addition to the BBM of early proximal convoluted tubuli, immunoreactivity is observed in the urinary pole of the Bowman's capsule, which is lined with cuboidal epithelial cells (arrows). (E) In the mouse small intestine tissue, where SGLT1/SLC5A1 is known to be expressed, SGLT2/SLC5A2 immunoreactivity is undetectable on the BBM of the columnar absorptive epithelial cells. (F) Immunoblot analysis of the membrane fraction of mouse kidney samples with the anti‐SGLT2/SLC5A2 antibody (Antigen −) depicts broad immunoreactive signal bands with molecular masses around 70 kDa, as well as relatively distinct bands with molecular masses of 72 and 70 kDa, and a distinct band with molecular mass of 26 kDa (arrows). Preabsorption of the antibody with recombinant SGLT2/SLC5A2 protein antigen (Antigen +) prevents the immunoreactive signal bands. Scale bars: 50 μm (A‐E).

We first confirmed the specificity of the SGLT2/SLC5A2 antibody used in this study. Immunohistochemical staining of human and mouse kidney samples with the anti‐SGLT2/SLC5A2 antibody revealed specific labeling on the BBM of the early proximal convoluted tubules (Fig. 1A,D). In mouse kidney sections, SGLT2/SLC5A2 immunoreactivity was observed not only in the PTE cells but also in the proximal tubule‐like cuboidal epithelial cells lining most renal corpuscles at the urinary pole (Fig. 1D, arrows), the staining pattern consistent with the observation in adult mice.^23^ Straight parts of proximal tubules (Fig. 1B, asterisks) and BBM of columnar absorptive cells of the small intestine (Fig. 1E), where SGLT1/SLC5A1 is known to be localized, were negatively immunostained with this antibody. Preabsorption of the antibody with recombinant SGLT2/SLC5A2 protein antigen completely abolished the immunoreactivity in the PTE cells of human kidney samples (Fig. 1C).

Murine SGLT2/SLC5A2 is composed of 670 amino acids, with a predicted molecular mass of 73 kDa.^24^ Immunoblot analysis of the membrane fraction of mouse kidney samples with the anti‐SGLT2/SLC5A2 antibody revealed broad immunoreactive signal bands with a molecular mass of approximately 70 kDa. Moreover, we observed relatively distinct bands with a molecular mass of 72 and 70 kDa, and a distinct band with molecular mass of 26 kDa (Fig. 1F lane Antigen −, indicated by arrows). Pre‐absorption of the anti‐SGLT2/SLC5A2 antibody with recombinant SGLT2/SLC5A2 protein antigen resulted in abolition of all the immunoreactive bands (Fig. 1F: lane Antigen +).

These results suggest that the antibody used in this study recognizes a protein consistent with SGLT2/SLC5A2 in human and mouse tissues in terms of its immunohistochemical localization and molecular mass, with a minimal possibility of crossreactivity to SGLT1/SLC5A1.

### Immunohistochemical observations of SGLT2/SLC5A2 in autopsied human brains

**Figure 2:**
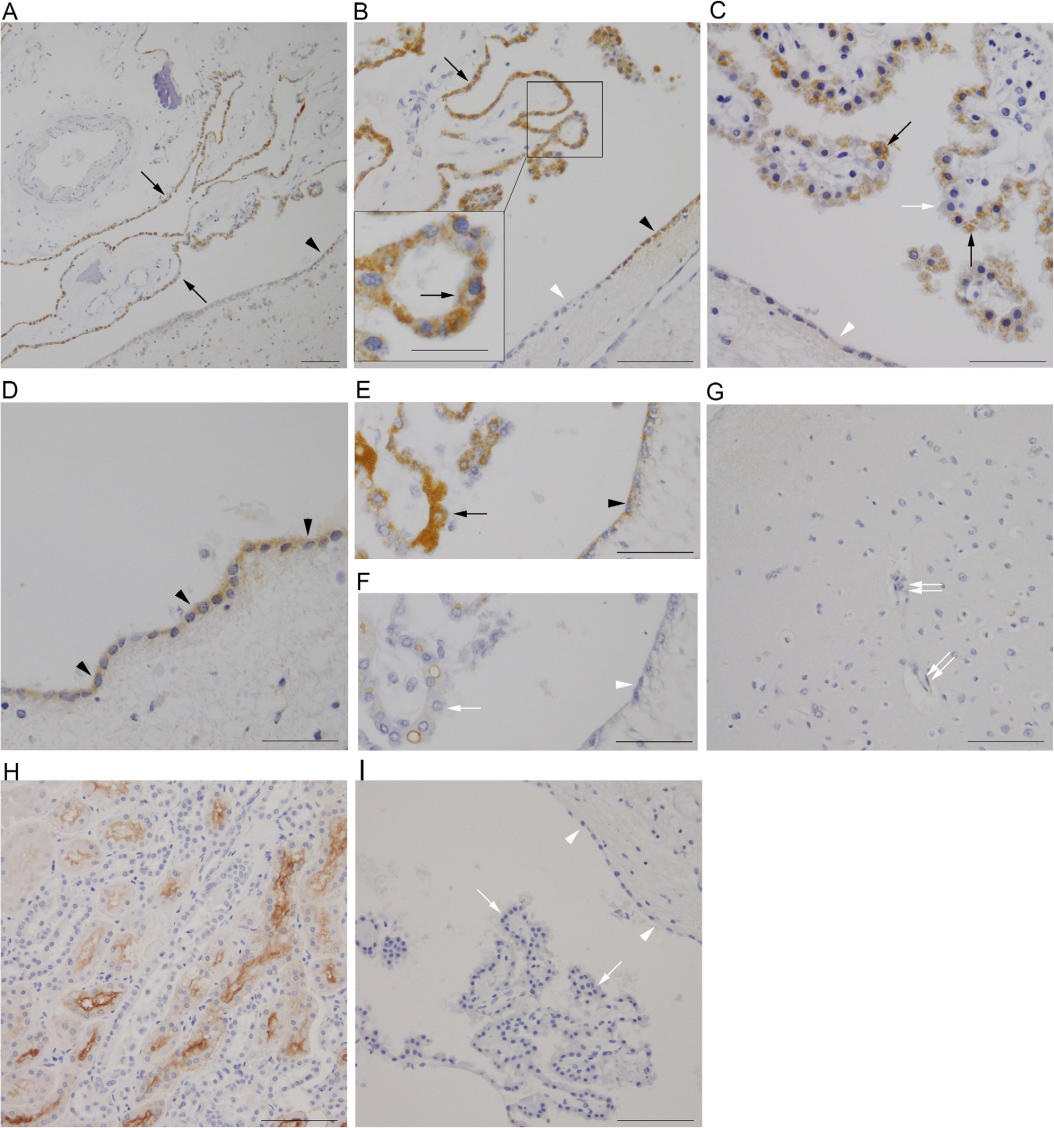
Immunohistochemical observations of SGLT2/SLC5A2 (A–G) and SGLT1/SLC5A1 (H, I) in human brain (A‐G, I) and kidney (H) tissues. (A–D) CPE cells (arrows, A–C) and ependymal cells (arrowheads) (A, B, D) show granular immunoreactivity for SGLT2/SLC5A2 in the cytoplasm. Inset in (B) indicates a magnified image of SGLT2/SLC5A2‐positive CPE cells. Some CPE (open arrow) (C) and ependymal cells (open arrowheads) (B, C) are negative for SGLT2/SLC5A2. (E, F) SGLT2/SLC5A2 immunohistochemistry without preabsorption reveals immunoreactivity in the cytoplasm of CPE (arrow) and ependymal (arrowhead) cells (E). Preabsorption of the anti‐SGLT2/SLC5A2 antibody with the recombinant antigen abolishes immunoreactivity in CPE (open arrow) and ependymal (open arrowhead) cells (F). (G) The immunoreactivity is undetectable in neurons, glia, and endothelial cells (double open arrows) in the lateral occipitotemporal cortex. (H) Immunohistochemical staining of human kidney samples with the anti‐SGLT1/SLC5A1 antibody reveals immunoreactivity on the BBM of the straight part of proximal tubuli. (I) SGLT1/SLC5A1 immunoreactivity is undetectable in CPE (open arrows) and ependymal (open arrowheads) cells. Case 1 (E–G), Case 2 (A), Case 3 (B), Case 4 (C, D, H, I). Scale bars: 100 μm (A, B, G, H, I), 20 μm (inset, B), 50 μm (C–F).

**Figure 3:**
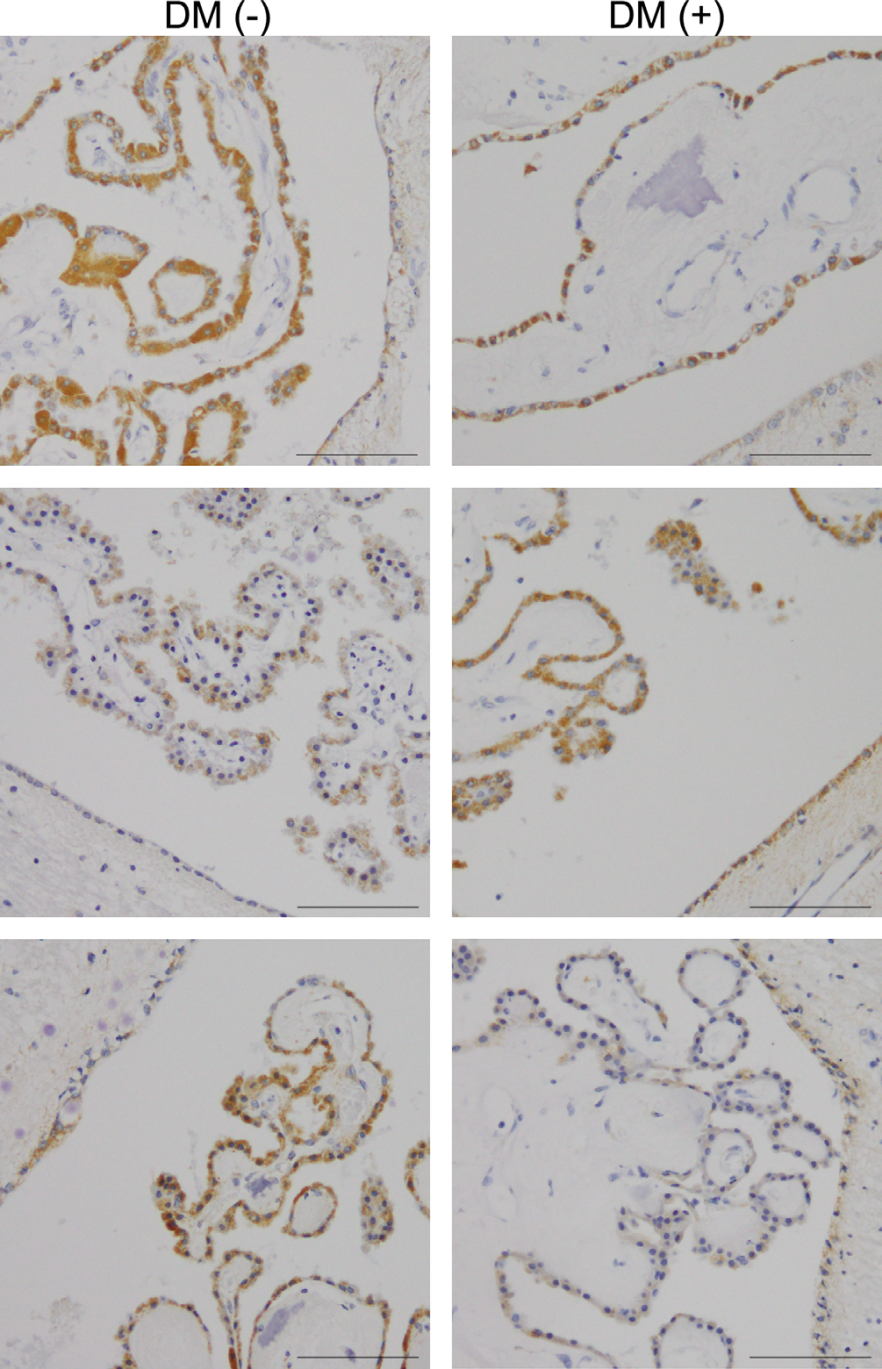
Representative views of SGLT2/SLC5A2 immunohistochemistry in autopsied human brains from all subjects examined. To compare the effect of DM on the immunoreactivity in CPE (CPE) and ependymal cells, results from subjects with or without DM (DM [+] and DM [−], respectively) are presented adjacently. Scale bars: 100 μm.

Immunohistochemical staining with the anti‐SGLT2/SLC5A2 antibody in human brain samples revealed a heterogeneous granular immunoreactivity in the cytoplasm of CPE and ependymal cells in all subjects (Figs 2A–D; 3). Most of the CPE cells were immunopositive for SGLT2/SLC5A2, although signal intensity was heterogeneous from cell to cell and from subject to subject (Figs 2A–C; 3). By contrast, fewer than half of the ependymal cells exhibited relatively weak immunoreactivity for SGLT2/SLC5A2 in all subjects (Fig. 2A–D). Immunoreactivity on CPE and ependymal cells was abolished by preincubating the SGLT2/SLC5A2 antibody with recombinant antigen (Fig. 2E, F). Neurons, glia, and endothelial cells in the cerebral neocortex and hippocampus showed no significant SGLT2/SLC5A2 immunoreactivity (Fig. 2G). The presence of DM seemed to have little impact on SGLT2/SLC5A2 immunoreactivity in the CPE and ependymal cells (Fig. 3).

Immunohistochemical staining with the anti‐SGLT1/SLC5A1 antibody revealed a clear immunoreactivity on the BBM of the straight part of the proximal tubule (Fig. 2H), whereas it was undetectable in the CPE and ependymal cells. (Fig. 2I).

### Expression status of SGLT2/SLC5A2 in mouse brains

**Figure 4:**
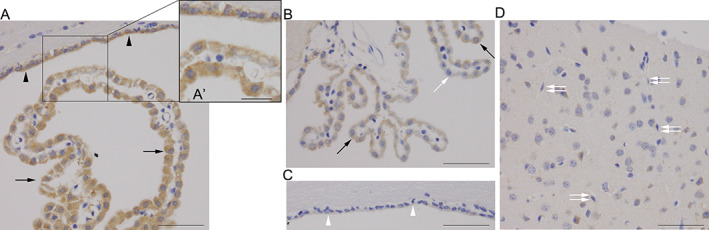
Immunohistochemical observations of SGLT2/SLC5A2 in mouse brains. (A) Granular heterogeneous immunoreactivity is observed in the cytoplasm of CPE (arrows) (A, B) and ependymal (arrowheads) cells. (A′) A magnified image shows SGLT2‐positive CPE and ependymal cells in the indicated area of (A). (B, C) A part of CPE (open arrow) (B) and ependymal (arrowheads) cells (C) does not show immunoreactivity for SGLT2/SLC5A2. (D) In the cerebral cortex, neurons, glia, and endothelial cells (open double arrows) show very weak or no immunoreactivity for SGLT2/SLC5A2. Scale bars: 50 μm (A‐D), 20 μm (A′).

SGLT2/SLC5A2 immunohistochemistry in mouse brains revealed a granular immunoreactivity in the cytoplasm of CPE and ependymal cells (Fig. 4A, A′, B). The intensity of SGLT2/SLC5A2 immunoreactivity in these cells showed heterogeneity (Fig. 4A–C), consistent with results from human brains. Cells in the cerebral neocortex and hippocampus, including neurons, glia, and endothelial cells, exhibited very weak or no SGLT2/SLC5A2 immunoreactivity (Fig. 4D). Immunohistochemical staining of mouse brains with the anti‐SGLT1/SLC5A1 antibody revealed no immunoreactivity in CPE and ependymal cells (data not shown).

**Figure 5:**
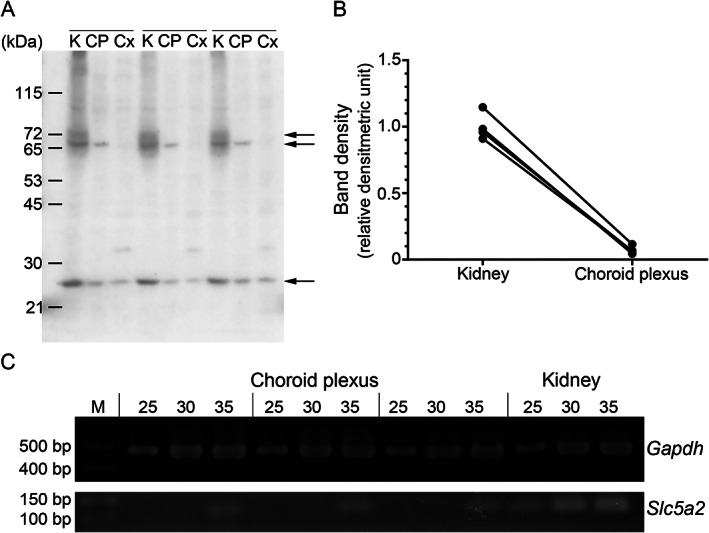
Expression of sodium/glucose cotransporter 2 (SGLT2)/solute carrier family 5 member 2 antibody (SLC5A2) protein and mRNA in mouse choroid plexus. (A) Immunoblot analysis of the membrane fraction of mouse kidney (K), choroid plexus (CP) and cerebral cortex (Cx) with anti‐SGLT2/SLC5A2 antibody. Results from three mice are presented, with samples of different tissues from each mouse arranged in consecutive lanes (indicated by lines above lanes). Results of immunoblotting (A) and densitometry (B) of SGLT2/SLC5A2 protein and RT‐qPCR of *GAPDH* and *SLC5A2* mRNA (C) in mouse brains and kidneys. (A) Immunoblot analysis was performed using the membrane fraction of mouse kidney (K), choroid plexus (CP), and cerebral cortex (Cx). Results from three mice are presented, with samples of different tissues from each mouse arranged in consecutive lanes (indicated by lines above lanes). Arrows on the right side of the blot indicate the position of distinct immunoreactive bands at 72, 70, and 26 kDa, respectively. (B) Immunoreactive signal bands with molecular mass around 70 kDa in mouse kidney and choroid plexus are quantified by densitometry. Results from four mice are analyzed and data from a single mouse are presented as a pair of dots connected with a line. (C) Expression status of *Slc5a2* and *Gapdh* mRNA in mouse choroid plexus is compared between choroid plexus and kidne tissues. M, DNA molecular weight marker.

Immunoblot analysis of the membrane fraction of mouse kidney, choroid plexus, and cerebral cortex with the anti‐SGLT2/SLC5A2 antibody revealed different band patterns. Diffuse immunoreactivity around 70 kDa was observed in the kidney, while a sharp band was detected in the choroid plexus (Fig. 5A). By contrast, no signal around 70 kDa was detected in the cerebral cortex. The immunoreactive signal bands at 26 kDa were observed in all tissues (Fig. 5A). Densitometric analysis of the 70‐kDa bands in the kidney and choroid plexus revealed that SGLT2/SLC5A2 protein levels in the choroid plexus were 4.5–10.1% of those in the kidney of the same individual (*P* = 0.0002) (Fig. 5B).

RT‐PCR analysis revealed that *Slc5a2*‐specific amplicons appeared after 25 and 35 reaction cycles in the kidney and choroid plexus, respectively (Fig. 5C). Sequencing analysis of the amplicons confirmed them as being the expected fragments of mouse *Slc5a2* (data not shown).

## DISCUSSION

SGLT2/SLC5A2 protein has been considered to exclusively localize in the BBM of PTE cells, implying that SGLT2‐Is exert their direct pharmacological effect solely through the kidney. The results presented here suggest that SGLT2/SLC5A2 is also expressed in CPE and ependymal cells along with PTE cells.

The lack of commercially available well‐characterized antibodies has hampered comprehensive studies to map SGLT expression in human tissues.^16^ Our validation experiments revealed that the antibody used in this study recognized an antigen compatible with SGLT2/SLC5A2 in terms of localization and molecular mass. SGLT2/SLC5A2 has 59% amino acid sequence identity to SGLT1/SLC5A1.^25^ However, there is little possibility of cross‐reactivity, because the amino acid sequence of the antigen protein (amino acid residues 220–266 of human SGLT2/SLC5A2) has no overlap with that of human SGLT1/SLC5A1. Moreover, the BBM of the straight part of proximal tubules and intestinal epithelial cells, where SGLT1/SLC5A1 is expressed, were negatively stained with the antibody used (Fig. 1B, E), further highlighting its specificity. The slight discrepancy between the observed and predicted molecular mass (73 kDa) may be explained by the fact that membrane proteins migrate faster than expected on SDS‐PAGE gels, with diffuse bands presumably due to incomplete denaturing by SDS.^16^ Although the identity of 26‐kDa bands is uncertain, we detected them in the membrane fraction of other tissues, including the cerebral cortex (Fig. 5A), liver, spleen, pancreas, lung, and small intestine (data not shown). This band may represent a degradation product of SGLT2/SLC5A2 protein, because it disappears after preincubation of the antibody with the antigen protein (Fig. 1F); however, it may also result from a nonspecific reaction.

Our immunohistochemical study revealed heterogeneity in the SGLT2/SLC5A2 immunoreactivity in CPE and ependymal cells (Figs 2, 3, 4). Although the reason for this heterogeneity remains unknown, diversity in the ultrastructure and molecular expression status among CPE and ependymal cells has been reported.^26^, ^27^, ^28^ In our previous study, we also reported focal immunoreactivity for low‐density lipoprotein receptor in ependymal cells along with a weak and ununiform immunoreactivity for insulin‐degrading enzyme in CPE and ependymal cells in autopsied human brains.^22^ Each CPE and ependymal cell might have an individualized molecular expression profile to fulfill the function of choroid plexus and ependyma as a whole.

Previous studies have shown that the expression of SGLT2/SLC5A2 is increased in renal PTE cells from human subjects with T2DM.^29^, ^30^ By contrast, our immunohistochemical study showed no apparent differences in SGLT2/SLC5A2 expression in CPE and ependymal cells between subjects with and without DM (Fig. 3). This discrepancy could be attributed to the small number of subjects and the lack of detailed information regarding the clinical history of DM. Further investigation is, thus, warranted to determine the effect of DM on the expression of SGLT2 in CPE and ependymal cells.

Our immunoblot analysis has disclosed that SGLT2/SLC5A2 proteins were enriched in the membrane fraction (Fig. 5A), while immunohistochemistry revealed cytoplasmic granular immunoreactivity in CPE and ependymal cells (Figs. 2A–E; 3; 4A, B). These observations are apparently inconsistent with the plasma membrane localization of SGLT2/SLC5A2 in PTE cells. Because our membrane fraction samples contain intracellular membranes, SGLT2/SLC5A2 may localize in the organelle's membranes and catalyze the transport of sodium/glucose through intracellular membranes. Similar subcellular localization has been reported for GLUT8, a glucose/fructose transporter which has been found to be associated with the plasma membrane of endosomes, lysosomes, and endoplasmic reticulum.^31^ The variation in posttranslational modifications like glycosylation may explain the difference in subcellular localization. In the case of GLUT4, *N*‐glycosylation contributes to the stability of the newly synthesized protein and translocation from intracellular vesicles to the cell surface in response to insulin treatment.^32^ We evaluated whether different band patterns, around 70 kDa, between the kidney and choroid plexus resulted from the difference in the degree of glycosylation, but we were unable to find evidence regarding this (data not shown).

We recently reported that URAT1 and GLUT9, which are located on the apical and basolateral sides of PTE cells, respectively, are also present in CPE cells with inverted submembrane localization.^20^ The present findings provide another example indicating the similarities in the expression profile of transporter proteins between PTE and CPE cells.

A limitation of this study is the lack of functional data for SGLT2/SLC5A2 in CPE and ependymal cells. SGLT2/SLC5A2 is a low‐affinity/high‐capacity glucose transporter, and its function in the kidney is to reabsorb the majority of filtered glucose, at approximately 90%, in the early proximal tubules.^7^ Due to apparent intracellular localization, it is uncertain whether SGLT2/SLC5A2 plays the same role in CPE and ependymal cells as in PTE cells. In addition to glucose transporters, SGLTs may act as urea and water channels, Na^+^ uniporters, or glucose sensors.^16^ Future studies using cultured CPE cells and SGLT2/SLC5A2 knockout/mutant mice, especially CPE‐specific conditional knockout mice, may clarify the SGLT2/SLC5A2 function in these cells.

Despite the limitation mentioned above, SGLT2/SLC5A2 expression in CPE and ependymal cells implies that SGLT2‐Is may modulate the production of CSF and/or its glucose/electrolyte composition. Provided that SGLT2‐Is exhibit beneficial effects through the modulation of the CNS microenvironment, these inhibitors may contribute to reducing the risk of AD developing in T2DM patients, in addition to their therapeutic use as dual inhibitors of SGLT2 and AChE.^9^, ^10^ The influences of SGLT2‐Is use on brain functions and AD progression deserve further investigations for the better care and safety of DM patients.

## DISCLOSURE

Authors declare no conflict of interests for this article.
